# Insulin glycation by methylglyoxal results in native-like aggregation and inhibition of fibril formation

**DOI:** 10.1186/1471-2091-12-41

**Published:** 2011-08-05

**Authors:** Luis MA Oliveira, Ana Lages, Ricardo A Gomes, Henrique Neves, Carlos Família, Ana V Coelho, Alexandre Quintas

**Affiliations:** 1 Centro de Investigação Interdisciplinar Egas Moniz, Instituto Superior das Ciências da Saúde Egas Moniz, Campus Universitário, Monte da Caparica 2829-511 Caparica, Portugal; 2 Centro de Química e Bioquímica, Departamento de Química e Bioquímica, Faculdade de Ciências da Universidade de Lisboa, Edifício C8, 1749-016 Lisboa, Portugal; 3 Departamento de Análises Clínicas e Saúde Pública, Escola Superior de Saúde Dr. Lopes Dias, Instituto Politécnico de Castelo Branco, Campus da Talagueira 6000-767 Castelo Branco, Portugal; 4 Instituto de Tecnologia Química e Biológica. Universidade Nova de Lisboa, 2780-901 Oeiras, Portugal

## Abstract

**Background:**

Insulin is a hormone that regulates blood glucose homeostasis and is a central protein in a medical condition termed insulin injection amyloidosis. It is intimately associated with glycaemia and is vulnerable to glycation by glucose and other highly reactive carbonyls like methylglyoxal, especially in diabetic conditions. Protein glycation is involved in structure and stability changes that impair protein functionality, and is associated with several human diseases, such as diabetes and neurodegenerative diseases like Alzheimer's disease, Parkinson's disease and Familiar Amyloidotic Polyneuropathy. In the present work, methylglyoxal was investigated for their effects on the structure, stability and fibril formation of insulin.

**Results:**

Methylglyoxal was found to induce the formation of insulin native-like aggregates and reduce protein fibrillation by blocking the formation of the seeding *nuclei*. Equilibrium-unfolding experiments using chaotropic agents showed that glycated insulin has a small conformational stability and a weaker dependence on denaturant concentration (smaller m-value). Our observations suggest that methylglyoxal modification of insulin leads to a less compact and less stable structure that may be associated to an increased protein dynamics.

**Conclusions:**

We propose that higher dynamics in glycated insulin could prevent the formation of the rigid cross-β core structure found in amyloid fibrils, thereby contributing to the reduction in the ability to form fibrils and to the population of different aggregation pathways like the formation of native-like aggregates.

## Background

Insulin is a small protein hormone that is crucial for the control of glucose metabolism. It regulates blood glucose levels by indirectly stimulating glucose transport across the cell membrane and by down regulation of enzymes involved in gluconeogenesis. External administration of insulin is critical in *Diabetes *type I, where autoimmune response causes a progressive and permanent destruction of the insulin-producing cells in the pancreas due to an interplay of environmental and genetic factors [1-3]. Insulin is composed of two polypeptide chains, the A-chain (21 residues) and the B-chain (30-residues) linked together by two disulfide bonds [4,5]. In the secretory vesicles of the pancreas the predominant form of insulin is a zinc-coordinated hexamer, formed by the association of three dimers, and stabilized by two to four zinc ions. However, when released into the blood stream, insulin is present in its biologically active form, *i. e*. the monomer [6,7]. Monomeric insulin is an amyloid protein forming amyloid-like fibrils *in vitro*, which are promoted by elevated temperatures, low pH, and increased ionic strength [8,9]. Insulin amyloid-like fibrils are the hallmark of a clinical condition observed in insulin-dependent diabetic patients, called insulin injection amyloidosis [10]. In this pathological condition, full-length insulin molecules are found in fibrillar form at the site of frequent insulin injections [9,11,12]. Additionally it was recently shown that serum samples from Parkinson's disease patients display an autoimmune response to insulin oligomers and fibrils [13], possibly indicating the presence of insulin aggregates in this disease as well. Insulin fibril formation has also been a limiting factor in long-term storage of insulin for treatment of diabetes. Thus, better understanding of insulin fibrillation mechanisms could lead to new therapeutic strategies, safer handling and more cost-effective storage of insulin. Upon fibrillation, insulin undergoes structural changes from a predominantly α-helical state to a β- sheet rich conformation. The α- to β-transition appears only to occur upon fibril assembly [14], and recently Vestergaard and co-workers proposed that insulin oligomers have an overall helical shape [15]. Being intimately related with glycaemia, it is likely that insulin may be modified by reactive α-ketoaldehydes such as 3-deoxyglucosone, glyoxal and methylglyoxal. These highly reactive compounds have been considered the most accountable for toxicity at high glucose concentrations [16]. In fact, hyperglycemia induces the glycation of insulin in pancreatic β cells [17] and glycated insulin is unable to regulate glucose homeostasis in vivo and to stimulate glucose transport and adipose tissue lipogenesis [17]. Protein glycation is a *post*-folding modification whereby amino groups in lysine and arginine side chains react irreversibly with carbonyl molecules forming advanced glycation end-products (AGE). Glycation exerts profound effects on protein structure, stability and function. AGE formation in proteins is associated to the clinical complications of diabetes *mellitus *[18], cataracts [19], uraemia [20], atherosclerosis [21] and age-related disorders [22]. Glycated proteins are present in β-amyloid (Aβ) deposits in Alzheimer's disease [23-25], in Lewy inclusion bodies of α-synuclein in Parkinson's disease [26] and in transthyretin amyloid deposits in familial amyloidotic polyneuropathy (FAP) [27]. In all these amyloid pathologies, β-sheet fibril structure and the presence of AGE are common features, suggesting a possible role for glycation in amyloid formation pathogenesis. Methylglyoxal is the most significant glycation agent in vivo, being one of the most reactive dicarbonyl molecules in living cells. This compound is an unavoidable by-product of glycolysis, arising from the non-enzymatic β-elimination reaction of the phosphate group of dihydroxyacetone phosphate and _D_-glyceraldehyde 3-phosphate [28]. Methylglyoxal irreversibly reacts with amino groups in lipids, nucleic acids and proteins, forming methylglyoxal-derived advanced glycation end-products (MAGE). In Aβ, glycation by methylglyoxal promotes the formation of β-sheets, oligomers and protofibrils and also increases the size of the aggregates [29]. Argpyrimidine is a specific methylglyoxal modification occurring in arginine residues, and was associated with amyloid diseases [27]. However, little is known about the effects of methylglyoxal glycation on the fibrillation of insulin. The aim of this work is to detail the molecular mechanisms of insulin fibril formation in the presence of methylglyoxal, which may be related to insulin toxicity and/or malfunction. We analyzed the effects of methylglyoxal on the structure, stability and fibrillation of insulin in a concentration-dependent manner. Full glycation pattern analysis of insulin showed that a single residue modification reduces insulin fibrillation by blocking the formation of the seeding *nuclei *and that by contrast, methylglyoxal glycation stabilizes soluble aggregates that retain native-like structure as showed by circular dichroism experiments.

## Results

### Characterization of insulin glycation by methylglyoxal

**Figure 1:**
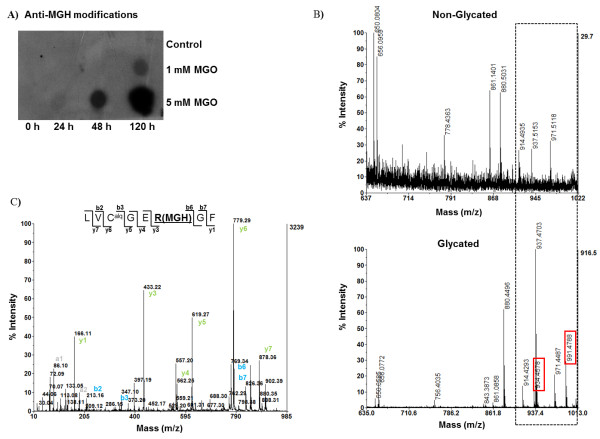
**Detection and location of MAGE-modified peptides**. (A) Dot-blot analysis with a specific antibody towards methylglyoxal-derived glycation adducts. A dose and time-dependent glycation is clearly detected. (B) The panels show representative sections of the MALDI-TOF/TOF spectra of peptides from unmodified and glycated insulin. New *m/z *peaks, absent from the control, are clearly detected in the mass spectra of the glycated insulin (highlighted in red). These new *m/z *values correspond to the mass of an insulin peptide plus the mass increment characteristic of a hydroimidazolone modification (54 Da). These peptides were analyzed by MS/MS, confirming the glycation of the arginine residue 46. (C) MS/MS spectrum of a glycated insulin peptide with *m/z *991.4788, showing the y and b fragment ions. The detected fragment ions arise from the amino acid sequence LVC^ALQ^GERGF, with a hydroimidazolone modification on the arginine residues. All the reported glycated peptides were confirmed by MS/MS data.

Prior to mass spectrometry analysis, non-glycated and glycated insulin were probed using a specific antibody towards methylglyoxal-derived glycation adducts. As shown in Figure 1A, a dose and time dependent glycation is clearly detected. To unequivocally identify glycated peptides and amino acid residues, non-glycated and glycated insulin were digested using chymotrypsin followed by MS and MS/MS analysis. A modified glycated peptide should be exclusively present in the MS spectrum of glycated insulin with a mass value corresponding to the insulin peptide plus the specific mass increment characteristic of a MAGE modification (72 Da for the lysine specific MAGE CEL and 54, 80 and 144 Da for the arginine-specific MAGE hydroimidazolones, argpyrimidine and tetrahydropirimidine respectively). This information was used to construct an inclusion list of modified peptides to be fragmented by an additional MS/MS experiment using the MALDI-TOF/TOF instrument. The sequence information thus obtained allowed the unequivocal identification of MAGE-modified peptides and also assignment of specific modified amino acid.

A comparative analysis of peptide mass spectra from the glycated and unmodified insulin reveals noticeable differences with several new peptides appearing exclusively in the glycated insulin (Figure 1B and Table 1). To identify MAGE-modified peptides and assign the glycated amino acid residues, the theoretical digestion was performed considering up to three chymotrypsin misscleavages (PeptideMass, Expasy, http://www.expasy.ch/tools/peptide-mass.html) and added to the resulting peptide masses the mass increment imposed by a MAGE modification (72, 54, 80 and 144 Da). Using this approach, several peptides, appearing only in the peptide mass spectrum of glycated insulin with a specific MAGE mass increment were observed (Figure 1B). For example, the species at *m/z *of 991.4788 may correspond to the B-chain peptide 41-48 (LVC^a|q^GERGF) with *m/z *937.4603 plus 54.018 Da, a mass increase characteristic of a hydroimidazolone (MGH) modification. This strongly suggests that the arginine residue 46 is glycated by methylglyoxal with the formation of a hydroimidazolone. In agreement, the observed peptide with an *m/z *934.4578 corresponds to the same peptide with a hydroimidazolone at R46 but without cysteine alquilation (C^a|q^). To unequivocally confirm these data, MS/MS experiments were performed to provide sequence information. When using the CID fragmentation technique, bond breakage mainly occurs through the lowest energy pathway, that is, the peptide bond, leading to b-ions (when the charge is retained by the amino-terminal fragment) or y-ion (when it is retained by the carboxy-terminal fragment). Thus, if an amino acid residue is modified, the particular y and complementary b ions, which encompasses the modification, will have the particular amino acid mass value plus 54.018 Da for hydroimidazolone. Taken the peptide with *m/z *of 991.4788 [LVC^a|q^GER(MGH)GF] (Figure 1C), we observed that the mass difference between y_1 _and y_2 _ions corresponds to an F residue, and the mass difference between y_2 _and y_3 _ions corresponds not to the addition of an G and R residue (57 + 156 Da) but to the addition of G and R residue plus the MGH modification on arginine (267 Da in total). The remaining mass differences between consecutive y ions also show this mass increment. The same feature is observed for the b ions. This clearly confirms that the amino acid residue R46 is modified by methylglyoxal. Only modified amino acid residues with confirmed sequence information were considered. Results are summarized in Table 1.

**Table 1:** Assignment of glycated amino acid residues.

Observed mass (Da)	Theoretical mass (Da)	Peptide sequence	Mass Increase (Da)	MAGE	Glycated residue
934.496	880.435	LVCGE**R**GF (41-48)	54.061	MGH	R46

991.521	937.456	LVC*GE**R**GF (41-48)	54.040	MGH	R46

1138.590	1084.524	LVCGE**R**GFF (41-49)	54.066	MGH	R46

1171.590	1027.503	LVCGE**R**GFF (41-49)	144.087	THP	R46

1228.616	1084.524	LVC*GE**R**GFF (41-49)	144.092	THP	R46

In all cases, the observed peptide mass has a mass increment specific of a methylglyoxal-derived AGE modification. The specific MAGE are indicated in the table and the modified amino acid residues are highlighted in the peptide sequence. (MGH - hydroimidazolone; THP - tetrahydropyrimidine)

*peptide with cysteine alquilation.

In the end, only the arginine residue in insulin was found to be glycated with the formation of either hydroimidazolone or tetrahydropirimidine. Similar results were observed in a previous study that characterized methylglyoxal modification of insulin. In that study the modification of the arginine residue with a 54 Da mass increase was detected [30]. Even though authors claimed that this mass increase corresponds to a Schiff base formation, this mass increment is characteristic of an MGH advanced glycation end-product. The glycation reactions by methylglyoxal are very fast [31] so the formation of advanced glycation products are expected. In this work, we observed that the arginine residue may also be modified with the formation of a tetrahydropirimidine (mass increment of 144 Da). This result is in agreement with our previous data showing the inherent heterogeneity of *in vitro *methylglyoxal glycation reactions [32]. In contrast, no evidences of glycation in the N-terminal and the lysine residue were observed by our mass spectrometry analysis. Although the N-terminal of insulin was found to be the major glycation target when using glucose as glycation agent [33,34], it is well known that methylglyoxal preferentially reacts and modify arginine residues [35].

### Methylglyoxal reduces insulin fibril formation

**Figure 2:**
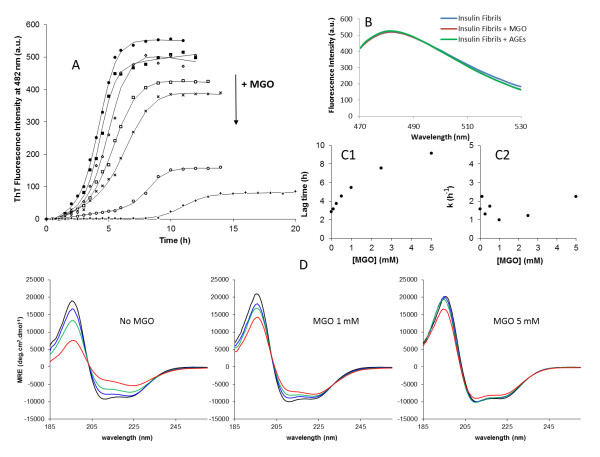
**Effect of methylglyoxal concentration on the kinetics of fibril formation of human insulin**. (A) Kinetics of fibrillation at different MGO concentrations monitored by ThT fluorescence. The symbols represent the average of ThT fluorescence intensities determined in three experiments, and the lines represent the best fit using the equation 1. Methylglyoxal concentrations used were 0 (•), 0.1 (^■^), 0.25 (◇), 0.5 (^□^), 1 (×), 2.5 (○) and 5 (+) mM. The decreasing in fluorescence intensities of the curves plateau are correlated with increasing methylglyoxal concentrations. (B) Evaluation of ThT quenching by methylglyoxal and AGEs. Non-glycated insulin fibrils were probed by ThT fluorescence after 8 h incubation (blue). Subsequently insulin fibrils were mixed with methylglyoxal (red) and glycated insulin containing AGEs (green) and probed again by ThT fluorescence. Fluorescence spectra show no quenching of ThT fluorescence induced by either methylglyoxal (red) or AGEs (green). (C) Dependence of the kinetic parameters lag time (C_1_) and apparent rate constant (C_2_) as a function of methylglyoxal concentration. Lag time is taken as x_0_-2τ and the *k *is given by 1/τ. (D) α- to β- transition of insulin at the indicated methylglyoxal concentrations during the fibrillation process followed by circular dichroism. CD spectra were collected at time 0 h (black), 3 h (blue), 5 h (green) and 7 h (red) incubation. Measurements were all performed at 37°C with agitation of the reaction mixture.

To investigate the effects of methylglyoxal on insulin fibril formation, insulin was incubated with methylglyoxal at different concentrations in the appropriate aggregation conditions described in the "Methods" section. The insulin fibrillation process as a function of time and methylglyoxal concentration was monitored by ThT fluorescence and circular dichroism (Figure 2). Methylglyoxal glycation of insulin resulted in a substantial dose-dependent decrease in ThT fluorescence intensity at the end of the fibrillation which is consistent with a reduced insulin fibril formation (Figure 2A). These differences were probed not to occur by ThT quenching caused by methylglyoxal or AGEs (Figure 2B). To further explore the biochemical mechanism on the inhibition of fibril formation by methylglyoxal glycation, a kinetic analysis was performed. The fibrillation kinetics represented in Figure 2A exhibit characteristic sigmoidal curves with an initial lag phase, a subsequent growth phase and a final equilibrium phase. Such curves are consistent with a nucleation-dependent polymerization model, in which the lag corresponds to the nucleation phase and the exponential part to fibril growth (elongation) [36-39]. Equation 1 was fitted to the experimental data and yielded values for the fibrillation lag time and for the apparent first-order rate constant (k_app_) of fibrillation [40,41]. The dependence of the kinetic parameters of fibrillation on methylglyoxal concentration is represented in Figure 2C1 and 2C2. Clearly, the lag time increases as a function of methylglyoxal concentration, changing from 2.8 h in unmodified insulin to 9.1 h upon methylglyoxal glycation. By contrast, no significant changes in the apparent rate constant of fibrillation were observed. These results show a longer nucleation phase which indicates that methylglyoxal glycation blocks the formation of the seeding *nuclei*, without changing the fibril elongation rate.

To detect changes in protein conformation during the fibrillation process, insulin fibril formation was monitored by circular dichroism (Figure 2D). Insulin presented a mainly α-helical secondary structure with spectral local minima at 222 and 208 nm and a positive band below 200 nm, which are characteristics of α-helical conformations (Figure 2 - time 0 h). CD spectra collected at several time points along the fibrillation pathway, showed that fibril formation is accompanied by a conformational transition, suggesting loss of α-helix and gain of β-sheet. This shift was most extensive when methylglyoxal was absent and decreases with methylglyoxal in a concentration-dependent manner. These results show that glycation preserves insulin native conformation, blocking the α-helix to β-sheet transition characteristic of amyloid fibril formation. This is in agreement with the reduction of fibril formation observed in ThT kinetic measurements and suggests that there is a structural inertia to conformational changes in glycated insulin that is responsible for blocking the seeding *nuclei *formation, leading to a reduced fibril formation.

### Methylglyoxal induces protein oligomerization

**Figure 3:**
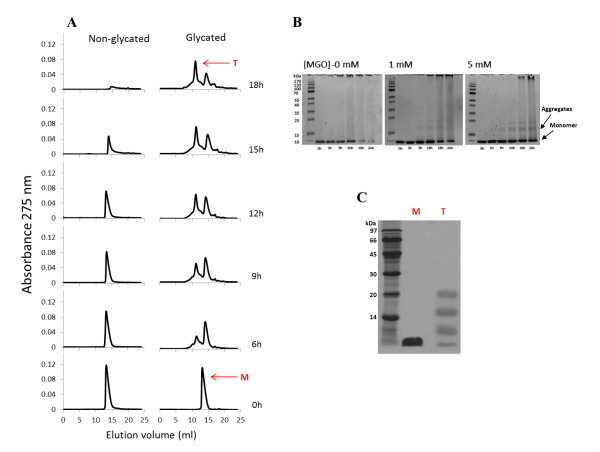
**Effects of methylglyoxal on the early steps of insulin aggregation**. Insulin (3 mg.ml^-1^) was incubated in the absence of methylglyoxal and in the presence of 5 mM of the glycation agent with stirring. Samples were collected at specific incubation times and immediately analysed by size exclusion chromatography (A) and PAGE (B). Sample buffer in PAGE did not contain SDS and β-mercaptoethanol in order to preserve the insulin oligomerization. To investigate the nature of insulin aggregates, the monomeric form of glycated insulin collected at incubation time 0 h (M) and the tetrameric form of glycated insulin collected at time 18 h (T) were analysed by a standard SDS-PAGE (C).

To investigate the early steps of protein aggregation, samples were collected at indicated incubation times and analyzed by size exclusion chromatography and PAGE (Figure 3). Non-glycated insulin appears as a single molecular species (elution volume of 14.04 ml) corresponding to the insulin monomer mass. No hexameric insulin species were detected confirming that the insulin sample preparation produced monomeric solution. The same feature was observed for glycated insulin at time 0 (elution volume of 13.68 ml), as it can be observed either by SEC or gel electrophoresis. The difference in the elution volumes is explained by an increased hydrodynamic radius of glycated insulin, which may be caused by a less compact structure formed upon glycation. During incubation time, the unmodified insulin monomer changes into amyloid fibrils. This can be observed from the native-PAGE (Figure 3B) where a reduction of insulin monomer (only species present at time 0 h) concomitant with the appearance of high molecular mass fibrils, unable to enter the separation gel, is clearly detected. Likewise, the insulin amyloid fibrils are unable to pass through the SEC column's filter and enter the stationary phase and thus a reduction of the SEC insulin monomer peak intensity with time is observed (Figure 3A). Interestingly, intermediate oligomeric species are apparently absent or in undetectable concentration. This may be due to the nature of soluble oligomers: they are intermediates of the aggregation process, and are therefore an extremely transient and labile species [42]. As soon as their concentration reaches a few percent, the oligomers are rapidly converted into amyloid fibrils with an organized β-structure. A very different scenario emerged when methylglyoxal is added. In this case, SEC peak intensity also becomes reduced, but other species are clearly detected on the chromatogram, corresponding to insulin soluble aggregates (Figure 3A). These aggregates are also observed in gel electrophoresis and show apparent molecular masses consistent with trimeric and tetrameric forms of insulin (Figure 3B). Moreover, high molecular mass species are only detected in the later incubation times compared to the control (without methylglyoxal). Taken together, these results show that methylglyoxal-induced glycation reduces insulin fibril formation and promotes the population of oligomeric states.

Protein glycation has been referred to induce protein aggregation due to cross-link formation [43,44]. However, when using methylglyoxal, only the lysine-lysine dimer MOLD is formed [45], which is a minor advanced glycation end-product compared to other AGE [46]. The fact that only a single arginine residue is glycated and that significant amounts of glycated insulin are in aggregated forms suggest that major non-covalent interactions are likely to be involved. The nature of the interactions in glycated insulin aggregates was evaluated by SDS-PAGE. The denaturing conditions of the SDS-PAGE induced significant dissociation of the glycated insulin tetramer (Figure 3C) showing that mainly non-covalent interactions are present in the insulin aggregates.

### Methylglyoxal effects on insulin structure and stability

**Figure 4:**
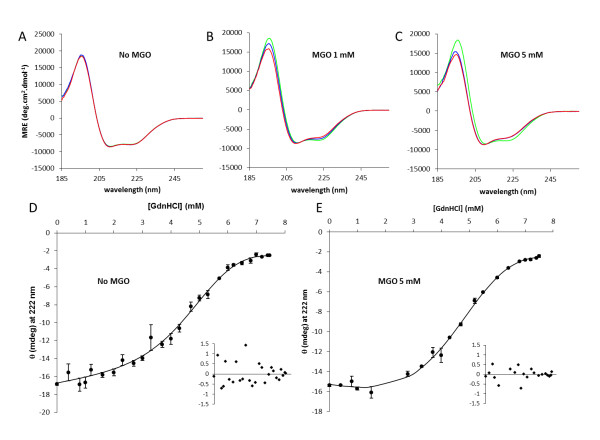
**Effects of methylglyoxal on insulin structure and stability**. Insulin (3 mg/ml) was incubated with 1 and 5 mM of methylglyoxal at 37°C without stirring for 48 h and compared with non-glycated insulin. Insulin secondary structure was monitored far-UV CD. Circular dichroism spectra were recorded as a function of time at different methylglyoxal concentrations (A - 0 mM; B - 1 mM; C - 5 mM). Spectra were collected at time zero (blue) and after 24 h (red) and 48 h (green) incubation. Deconvolution of the CD spectra are present in Table 2. Protein conformational stability was evaluated for native insulin (D) and glycated insulin (E) by guanidinium hydrochloride equilibrium denaturation curves at pH 7.4 and 37°C monitored by circular dichroism at 222 nm. The curves are non-linear least squares fits to a two-state unfolding model equation [71,72] representing the entire denaturation curve and using a linear extrapolation method to the experimental circular dichroism data [47]. The insets are the residues plot.

Our final set of experiments was aimed to investigate the structural changes imposed by methylglyoxal-derived glycation that might be associated to fibril inhibition and stabilization of oligomeric species. In these experiments insulin was incubated without agitation, a condition that does not promote aggregation, as observed by SEC experiments (see Additional File 1: Figure S1). In these conditions, insulin is glycated but remains almost entirely in monomeric form. In contrast with the results obtained when insulin was incubated in aggregation conditions, the CD spectra of non-glycated insulin remains unchanged during the incubation period (Figure 4A), while glycated insulin undergoes slight spectral changes (Figure 4B and 4C). Spectra deconvolution shows a redistribution of secondary structure elements in glycated insulin with a respective increase in β-sheet content, an increase in unordered structure and a reduction in the relative α-helical content (Table 2).

**Table 2:** Distribution of the structural element fractions for native and glycated insulin along time obtained by deconvolution of CD spectra using CDSSTR algorithm available on Dichroweb (Dichroweb; http://www.cryst.bbk.ac.uk/cdweb/html/home.html) [69,70].

[MGO] (mM)	Time (h)	α-Helix	β-Sheet	β-Turns	Unordered structure	NRMSD
0	0	31	23	22	24	0.028
	24	33	23	21	23	0.033
	48	32	22	22	24	0.029

1	0	31	24	21	24	0.027
	24	28	26	22	26	0.032
	48	24	27	22	27	0.036

5	0	32	22	22	24	0.022
	24	23	27	21	27	0.029
	48	23	28	21	27	0.035

The NRMSD parameter represents the normalized root mean square deviance.

We then assess the conformational stability of glycated and native insulin (Figure 4D and 4E). GdnHCl-induced denaturation was found to be reversible, as judged by CD experiments after dialysis of GdnHCl-denatured insulin (data not shown). Fits were made using the linear extrapolation method [47] in a non-linear least squares fitting procedure and yielded values for *ΔG^o^(H_2_O)*, the conformational stability, and *m*, the dependence of *ΔG^o^*on denaturant concentration. Table 3 shows the values obtained from the curves in Figure 4D and 4E for *ΔG^o^(H_2_O)*, *m*, and *Cm*, the denaturant concentration at the midpoint of the unfolding transition. Glycated insulin has a smaller conformational stability with *ΔG^o^(H_2_O) *of 2.66 ± 0,27 kcal.mol^-1 ^against 3,34 ± 0,33 kcal.mol^-1 ^for unmodified insulin. This decrease in conformational stability is also supported by the smaller *Cm *value of glycated insulin. In addition, glycation resulted in a weaker GdnHCl-dependence of unfolding (smaller *m*-value). The *m*-value has been correlated with the difference between accessible surface areas in the unfolded and folded states: *m *∝ ΔA, where ΔA = A_U _- A_N _[48]. This weak dependence may reflect a less compact folded structure or a more compact unfolded state. Putting these results together with the SEC experiments where glycated insulin has a small elution volume then the native insulin, suggest that the presence of a less compact structure is a more likely scenario, which may be the basis of a higher susceptibility to different unfolding and aggregation pathways.

**Table 3:** Thermodynamic parameters from GdnHCl unfolding studies of native and glycated insulin.

	*ΔG^o^*(H_2_O) (kcal·mol^-1^)	*m* (kcal·mol^-1^.M^-1^)	*Cm* (M)
Insulin	3.34 ± 0.33	0.63 ± 0.10	5.31 ± 0.98
Glycated Insulin	2.66 ± 0.27	0.52 ± 0.09	5.10 ± 0.98

Parameters were obtained by a direct fit of the model equations to experimental data in Figure 4 D and E. *ΔG^o^(H2O) *is the protein conformational stability; *m *is the dependence of ΔG^o^on denaturant concentration; *Cm *is the denaturant concentration at the midpoint of the unfolding transition.

## Discussion

Insulin is a protein hormone that regulates glucose concentration in blood. It is intimately related with glycaemia and is vulnerable to glycation by glucose and other highly reactive carbonyls like methylglyoxal. Additionally, it has the ability to aggregate and form amyloid-like fibrils that are characteristic of a clinical condition called insulin injection amyloidosis [10]. In this work we have investigated the effects of methylglyoxal-modification of insulin on structural and fibril-forming properties. Mass spectrometry data showed that methylglyoxal specifically modifies a single arginine residue in the B-chain. This is in agreement with a previous study that observed a methylglyoxal-derived modification on the arginine residue of the B chain [30]. The glycation of insulin in our experimental conditions promoted the coexistence on insulin molecules with the arginine residue modified to a hydroimidazolone and to a tetrahydropirimidine modification. This heterogeneity in *in vitro *glycation was already observed [32]. No modification on the lysine residues and N-terminal were detected by our experimental approach. Insulin glycation by _D_-glucose also led to the coexistence of protein molecules glycated at different residues [34]. In opposition to our results, the N-terminus of both chains and the lysine residue 29 were modified upon glucose glycation. This difference is not surprising since it is well documented that methylglyoxal preferentially reacts and modifies arginine residues [35].

Previous reports showed that AGE modifications accelerated the fibrillation of several proteins and peptides including β-amyloid peptide, tau and albumin [49,50]. Additionally, AGE-modified proteins were detected in amyloid deposits from several amyloidosis such as Alzheimer's [24,51], Parkinson's [26,52] disease and FAP [27]. In contrast with those amyloidogenic proteins, modification of β-2-microglobulin and α-synuclein by different glycation agents resulted in inhibitory effects on the formation and extension of fibrils [53,54]. Our data also showed that insulin fibril formation is substantially reduced upon methylglyoxal modification. The observed differences might be a consequence of the inherent properties of the native structure of each protein, or differential structural changes induced by AGE modifications as result of different glycation agents. In most of the cases mentioned above, fibrillation enhancement is achieved by modifying amyloidogenic proteins with glycating sugars like glucose or fructose while small and highly reactive carbonyls like methylglyoxal are apparently more prone to reduce fibril formation. A good example comes from α-synuclein where glyoxal and methylglyoxal inhibit fibril formation [54] while _D_-ribose glycation does not [55]. This suggests that different glycation agents lead to specific structural constraints that have a major role in protein fibrillation kinetics.

Insulin offers a structural simplicity of two short polypeptide chains constrained by one intramolecular and two intermolecular disulphide bonds and has well-known molecular mechanisms of fibril formation [8,56]. The insulin B-chain segment with the sequence LVEALYL is the smallest segment in the basis of fibril assembly, being crucial to the cross-β spine of the insulin fibril [56]. In full-length insulin molecules, there must be conformational changes for the LVEALYL side chains of the segment to be exposed and to interact with each other [56]. However, insulin glycation leads to native-like aggregation, as showed by CD experiments. This suggests that glycation impairs insulin conformational alterations, causing the inhibitory effects observed in the fibrillation process. Moreover our kinetic analysis of insulin aggregation showed an increase in fibrillation lag time. The lag time can be used to monitor the nucleation phase prior to the exponential stage of fibril elongation. Increasing lag time indicates that methylglyoxal glycation inhibits the fibrillation process by blocking the formation of the seeding *nuclei*. Accordingly fibril formation is reduced due to lack of a critical concentration of seeds.

Despite the inhibition of fibril formation, size exclusion chromatography experiments showed that glycation induces insulin aggregation. However these aggregates are small, soluble, non-fibrillar and native-like in structure, and apparently are not a consequence of a covalent crosslinking of insulin monomers. This implies that aggregation of modified insulin is not a merely result of a chemical reaction, but an outcome of complex folding interactions that are established and populates an off-pathway to fibril formation. A subject of intense investigation is whether the amyloid fibril deposits or the prefibrillar aggregates, called protofibrils, are the most potent mediators of cell damage, cytotoxicity and neurotoxicity. The finding that the severity of cognitive impairment in protein misfolding diseases correlates with the levels of small oligomeric species and not with the large fibrillar species has led researchers to the conclusion that the soluble small aggregates are the primary cause of the pathological symptoms [57-60]. Moreover, accumulation of AGE-modified proteins has been related to cellular responses including oxidative stress and the release of pro-inflammatory cytokines mediated by AGE:RAGE interaction [61,62]. Therefore it will be interesting to evaluate the cytotoxicity of the insulin glycated aggregates.

In order to understand what structural restrictions could cause this behavior, we investigated the effects of methylglyoxal glycation on the structure and stability of insulin. Circular dichroism experiments showed that modified insulin has a small conformational stability and a slight increase in β-sheet content when compared to the unmodified protein. This lower conformational stability is accompanied by a weaker dependence of ΔG^o^on denaturant concentration which is related to a less compact native structure or a more compact unfolded state [48]. Size exclusion chromatograms of glycated insulin showed a slight decrease in retention time of the insulin monomer, supporting the idea of a less compact native structure. Although most of the proteins have well-defined structures, they are not static molecules. Proteins are dynamic entities and possess an inherent flexibility. Having a lower contribution of van der Waals interactions, it is likely to expect that a less compact structure may result in a more dynamic one. The term dynamics is used for intrinsic protein molecular motions, while the term flexibility is used for the ability of a protein to adapt its structure to external stimuli. Accordingly, proteins are flexible as a consequence of their dynamics, yet their dynamics do not automatically result in flexibility. We propose that higher dynamics in glycated insulin could lead to impairment of the formation of the rigid cross-β core structure found in amyloid fibrils, resulting in a higher susceptibility to different unfolding and aggregation pathways. In this case other aggregation pathways that preserve native-like structure and comparable dynamics, like the small and soluble aggregates of glycated insulin observed in size exclusion chromatography, could be more likely populated.

## Conclusions

Insulin is a nearly all-alpha protein playing a central role in blood glucose homeostasis and is associated with a medical condition termed insulin injection amyloidosis, characterized by the formation and deposition of amyloid fibrils from insulin. Due to its main physiological role, insulin is a target for glycation by methylglyoxal. Protein glycation mostly impairs protein functionality by changing protein structure and stability, and AGE-modified proteins have been related to cellular responses including oxidative stress and the release of pro-inflammatory cytokines. Glycation has been associated with human conformational diseases, such as Alzheimer's disease, Parkinson's disease and Familiar Amyloidotic Polyneuropathy, which are associated to the formation of amyloid fibrils. Our results show that glycation of insulin by methylglyoxal reduce insulin fibril formation and leads to the formation of insulin native-like aggregates. In addition they suggest that modification of insulin leads to a less compact and less stable structure that may be associated to an increased dynamics, preventing the formation of the rigid cross-β core structure found in amyloid fibrils. Overall the present study points that methylglyoxal adducts can trigger a drifting from an amyloid aggregation to a native-like aggregation pathway, a mechanism that might be important in the context of the amyloidogenicity of AGE-modified proteins involved in conformational diseases.

## Methods

### Insulin preparation and glycation

Insulin exists in solution as an equilibrium mixture of monomers, dimers, tetramers and hexamers, and possibly higher associated states, depending on concentration, pH, metal ions, ionic strength and solvent composition [63]. A solution containing only insulin in the monomeric form was prepared taking into account the fluctuation of its association states in different milieu conditions as described [64]. Briefly, human zinc-free insulin (Sigma) was dissolved in ultra-pure miliQ water to a final concentration of 6 mg.ml^-1 ^and acidified with H_3_PO_4 _to a pH of 5 in order to obtain monomeric insulin. Insulin at pH 5 was then incubated for 15 min at room temperature and protein concentration was determined by absorbance at 275 nm (ε_275 _= 4560 M^-1 ^cm^-1^) in a UV-Visible spectrophotometer Jasco V-530. Finally, insulin was neutralized to pH 7 with NaOH 0.1 M and diluted to a final concentration of 3 mg.ml^-1^. Insulin preparation was proven to be in the monomeric form after pH neutralization as evaluated from size exclusion chromatography and native-PAGE experiments as described below. Also circular dichroism experiments showed that no structural changes or unfolding occurred with pH variations. In all assay, monomeric insulin was prepared in exactly the same way.

For the methylglyoxal-derived glycation of insulin, the protein preparation (3 mg.ml^-1^) was incubated with methylglyoxal (at several concentrations ranging from 0.1 to 5 mM) (a kind gift from Dr. Carlos Cordeiro, Centro de Química e Bioquímica, FCUL, Lisbon, Portugal) in 50 mM potassium phosphate buffer, pH 7.4, supplemented with 150 mM of NaF, at 37°C in sterile conditions. Samples were collected at different incubation times for analysis with the maximum incubation time of 48 hours. Control samples were treated in the same way but without methylglyoxal addition. To evaluate the effects of methylglyoxal on insulin stability and secondary structure changes, samples were incubated without stirring, a condition that avoid fibril formation, producing only glycated insulin in the monomeric state. In contrast, for the oligomerization and fibrillation kinetic studies, samples were incubated with vigorous agitation. Aliquots were collected in sterile conditions at defined incubation times from 0 to 4 hours and immediately analyzed.

### Characterization of insulin glycation by methylglyoxal using mass spectrometry and dot-blot analysis

Dot-blot assay was performed using a specific monoclonal antibody towards methylglyoxal-derived glycation (a kind gift from Dr. Ram Nagaraj, Case Western University, Cleveland, OH, USA), using a 1:2000 dilution. Washes, secondary antibody and detection procedures were performed using the BM Chemiluminescence Western Blotting Kit (Pierce) following the manufacturer's instructions.

To characterize the protein modification and assign the amino acid residues modified by methylglyoxal, a chymotrypsin digestion of insulin was performed. Protein samples were reduced with 10 mM dithiothreitol in 100 mM NH_4_HCO_3 _buffer (pH 8.0) at 55°C for 1 h and alkylated with 55 mM of iodoacetamide in 100 mM NH_4_HCO_3 _buffer (pH 8.0) in the dark for 30 min. In solution digestion were performed with chymotrypsin (Promega) using 50:1 ratio of protein:protease in 100 mM Tris-HCl buffer (pH 7.8) containing 10 mM CaCl_2 _for 16 h. Protein digestion was stopped by the addition of formic acid [(final concentration of 1% (v/v)]. The obtained peptide mixture was purified and concentrated by solid-phase extraction using home-made R2 Pore microcolumns (Applied Biosystems) as previously described [65]. Peptide mixture were eluted directly onto the MALDI target plate with 0.5 μl of α-CHCA matrix (5 mg.ml^-1^) prepared in 50% (v/v) acetonitrile with 0.1% (v/v) formic acid. The mixture was allowed to air dry (dried droplet method). Sample peptides were analysed in a MALDI-TOF-TOF mass spectrometer 4800 plus (Applied Biosystems) in positive reflectron mode for peptide mass determination. The mass spectrometer was externally calibrated using 4700 Calibration Mix (Applied Biosystems). Mass spectra were collected in a result-independent acquisition mode, typically using 1000 laser shots per spectrum and a fixed laser intensity of 3500 V. The peptides of interest (*i.e*., having a mass consistent with the mass increment of the modifications by methylglyoxal) were selected for MS/MS experiments using Collision Induced Dissociation (CID), with 1 kV collision energy and an air pressure of 106 torr. Two thousand laser shots were collected for each MS/MS spectrum using a fixed laser intensity of 4500 V. Raw data were generated by the 4000 Series Explorer Software v3.0 RC1 (Applied Biosystems). The identification of MAGE-modified peptide and amino acid residues was further validated using Peaks Studio 4.5 software (Bioinformatic Solutions Inc.), combined with manual inspection of the assigned sequence.

### Analysis of insulin-fibril formation and fibrillation kinetics

To investigate the effects of MGO in insulin fibril formation, solutions of monomeric insulin (prepared as described above) were incubated with stirring at 37°C in the presence of methylglyoxal at 0, 0.1, 0.25, 0.5, 1.0, 2.5 and 5.0 mM. Fibril formation was monitored with thioflavin T (ThT) binding assay as previously described [65,66]. Briefly, aliquots of 5 μl were removed and added to 0.5 ml of 10 μM ThT in 50 mM sodium phosphate buffer (pH 7.4) at room temperature and immediately analyzed. Fluorescence measurements were performed using a Perkin Elmer LS50B spectrofluorimeter, in quartz cuvettes with 1 cm excitation light path. ThT fluorescence was recorded immediately after ThT binding from 470 to 530 nm with excitation at 450 nm, an increment of 0.5 nm, an integration time of 1 s and 5 nm slits for both excitation and emission. For each sample, the signal was obtained as the ThT intensity at 482 nm from which was subtracted a blank measurement recorded prior to addition of insulin to the ThT solution. To test if methylglyoxal alone or the derived insulin AGEs interfere with ThT fluorescence of insulin fibrils, non-glycated insulin fibrils were produced in vigorous agitation by incubating monomeric insulin preparation (3 mg.ml^-1^) in the absence of methylglyoxal for 8 h. ThT fluorescence was then determined for insulin fibrils alone, in the presence of methylglyoxal (5 mM), and also in the presence of methylglyoxal-glycated insulin (3 mg.ml^-1^) prepared with vigorous agitation as described above.

ThT fluorescence measurements were plotted as a function of time and equation 1 was fitted to the experimental data [40,41].(1)

where Y is the fluorescence intensity and x_0 _is the time to 50% of maximal fluorescence. The initial base line during the lag phase is described by y_i _+ m_i_x. The final base line after the growth phase had ended is described by y_f _+ m_f_x. The apparent first-order rate constant (k_app_) for the growth of fibrils is calculated as 1/τ, and the lag time is calculated as x_0_-2τ. This expression is unrelated to the underlying molecular events, but provides a convenient method for comparison of the fibrillation kinetics.

### Size-exclusion and PAGE experiments

Aggregation of human insulin upon methylglyoxal glycation was monitored by size exclusion chromatography (SEC) and Native-PAGE. Solutions of monomeric insulin were incubated and stirred at 37°C in the presence of methylglyoxal at 0, 1 and 5 mM. Samples were analyzed by SEC at defined incubation times, after filtration with a 0.2 μm Whatman filter. SEC was performed with HPLC Jasco PU-2080 Plus isocratic pump with an UV detector JASCO 2075. The mobile phase was 50 mM sodium phosphate buffer pH 7.4 with 150 mM NaF. Separation was achieved on a molecular exclusion analytical column (Amersham-Pharmacia Superdex™ 75 10/300 GL) at a flow rate of 0.4 ml/min. Eluting peaks were monitored at 275 nm. Insulin samples were also separated by Native and SDS-PAGE on a Bio-Rad Mini-Protean 3 system, using a 12% separation gel and a 4% stacking gel. On Native-PAGE all buffers were prepared without SDS addition. Proteins were stained with Comassie Brilliant Blue [67].

### Circular dichroism and conformational stability measurements

Secondary structure analysis was performed by far-UV (185-260 nm) CD in a Jasco J810 spectropolarimeter equipped with a temperature control unit Julabo F25 using an insulin concentration of 3 mg.ml^-1^. Far UV CD spectra were recorded with 0.01 cm (linear) path length quartz cuvette at 37°C in 50 mM sodium phosphate buffer pH 7.4 with 150 mM NaF. For each spectrum, three scans were averaged and protein concentration was determined by absorbance at 275 nm using the above mentioned insulin extinction coefficient in a UV-Visible spectrophotometer Jasco V-530. For protein secondary structure estimation, CD spectra were deconvoluted using the CDSSTR [68] deconvolution algorithm on Dichroweb [69,70]. CD spectra of the appropriate buffers were recorded and subtracted from the protein spectra.

CD denaturation curves for non-glycated and glycated insulin monomer were constructed using the ellipticity at 222 nm, monitored at 37°C after 24 h incubation with guanidinium hydrochloride (GdnHCl) at various concentrations. The denaturation of glycated and non-glycated insulin could be described as sigmoidal curves and were analyzed according to a two-state unfolding model M ↔ U using the linear extrapolation method [47] in a non-linear least squares fitting procedure and yielded values for ΔG^o^(H_2_O), the conformational stability, and m, the dependence of ΔG^o^on denaturant concentration. Cm, the denaturant concentration at the midpoint of the unfolding transition was calculated as *C_m_= G^o^(H_2_O)/m*. Denaturation curves for monomeric species were analyzed considering the equation developed by Santoro & Bolen [71,72].

## Abbreviations

α-CHCA: α-cyano-4-hydroxicinamic acid; Aβ: β-amyloid peptide; AGE: Advanced glycation end-products; CD: Circular dichroism; CID: Collision induced dissociation; FAP: Familial amyloidotic polyneuropathy; GdnHCl: Guanidinium hydrochloride; MALDI: Matrix-assisted laser-desorption ionization; MAGE: Methylglyoxal-derived advanced glycation end-products; MGH: Hydroimidazolone; MGO: Methylglyoxal; MOLD: Methylglyoxal lysine dimer; RAGE: Receptor for advanced glycation end-products; SEC: Size exclusion chromatography; THP: tetrahydropyrimidine; ThT: Thioflavin T; TOF: Time of flight.

## Authors' contributions

LMAO conceived the part of the study related with the fibrillation kinetics of insulin, carried out part of the experimental procedures and was mainly responsible for the experimental setup and data analysis as well as the initial drafting of the manuscript. AL is responsible for executing most part of the experiments. RAG conceived, executed and interpreted the experiments related with mass spectrometry and participated in the writing of the manuscript. HN executed the experiments associated to the thioflavin T curves. CF participated in the experiments to prove the nature of insulin aggregates. AVC contributed to this work with her expertise concerning mass spectrometry. AQ designed and coordinated the study and was mainly involved in the preparation and reviewing of the manuscript. All authors read and approved the final manuscript.

## Supplementary Material

Additional file 1 **Figure S1. Evaluation of insulin aggregation in non-stirring conditions**. Insulin incubation in 50 mM potassium phosphate buffer, pH 7.4 supplemented with 150 mM of NaF, at 37°C in sterile conditions without stirring. Gel filtration experiments show that insulin does not aggregate in this incubation conditions, remaining in the monomeric form. Click here for file
