# Supplementary figures and images for: Insulin glycation by methylglyoxal results in native-like aggregation and inhibition of fibril formation
Source: BMC Biochem. 2011 Aug 5;12:41. doi: 10.1186/1471-2091-12-41 (PMC3175161; doi:10.1186/1471-2091-12-41)

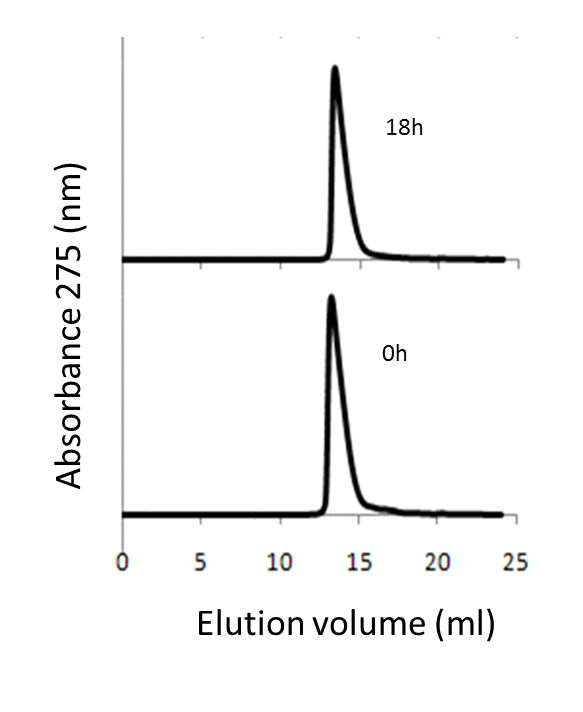

Supplement: Additional file 1 — Figure S1. Evaluation of insulin aggregation in non-stirring conditions. Insulin incubation in 50 mM potassium phosphate buffer, pH 7.4 supplemented with 150 mM of NaF, at 37°C in sterile conditions without stirring. Gel filtration experiments show that insulin does not aggregate in this incubation conditions, remaining in the monomeric form. [file 1471-2091-12-41-S1.TIFF]
